# The bacillary and macrophage response to hypoxia in tuberculosis and the consequences for T cell antigen recognition

**DOI:** 10.1016/j.micinf.2016.10.001

**Published:** 2017-03

**Authors:** Gareth Prosser, Julius Brandenburg, Norbert Reiling, Clifton Earl Barry, Robert J. Wilkinson, Katalin A. Wilkinson

**Affiliations:** a Tuberculosis Research Section, Laboratory of Clinical Infectious Diseases, National Institute of Allergy and Infectious Diseases, Bethesda, MD, 20892, United States; b Microbial Interface Biology, Priority Research Area Infections, Forschungszentrum Borstel, Leibniz Center for Medicine and Biosciences, Parkallee 1-40, D-23845, Borstel, Germany; c Clinical Infectious Diseases Research Initiative, Institute of Infectious Disease and Molecular Medicine, University of Cape Town, Observatory, 7925, South Africa; d The Francis Crick Institute, London, NW1 2AT, United Kingdom; e Department of Medicine, Imperial College, London, W2 1PG, United Kingdom; f German Center for Infection Research (DZIF), Partner Site Hamburg-Borstel-Lübeck, Borstel, Germany

**Keywords:** Tuberculosis, Hypoxia, T cells, Antigens, Macrophage, Lipid droplets

## Abstract

*Mycobacterium tuberculosis* is a facultative anaerobe and its characteristic pathological hallmark, the granuloma, exhibits hypoxia in humans and in most experimental models. Thus the host and bacillary adaptation to hypoxia is of central importance in understanding pathogenesis and thereby to derive new drug treatments and vaccines.

## 1 Introduction

Since tuberculosis (TB) was declared a global health emergency in 1993 [1] a number of important control efforts have led to a fall of TB-associated mortality and the saving of 45 million lives [2]. However, up to a third of the world's population is estimated latently infected with *Mycobacterium tuberculosis* (Mtb), serving as a reservoir for many of the estimated 9·6 million people who developed TB worldwide in 2014, leading to 1·5 million deaths. Thus, TB now ranks alongside HIV as a leading cause of death worldwide, and the rate of HIV-TB co-infection worldwide in 2014 was 12% [2].

Mtb is transmitted by the cough of an infected person (aerosolized) and inhaled into the alveoli of a new host. This process can lead to three possible outcomes: i) a minority develop active primary progressive TB disease and develop a detectable but ineffective acquired immune response (immune sensitization), ii) the majority develop latent TB infection that is contained throughout their life by an effective acquired immune response, and iii) a small proportion of those latently infected develop post-primary TB as a result of reactivation of their latent infection, which can be triggered by immune suppression such as HIV-1 infection [3]. Latent Mtb infection (LTBI) is defined solely by evidence of immune sensitization by mycobacterial proteins: a positive result in either the tuberculin skin test (TST) or an *in vitro* interferon gamma release assay (IGRA), in the absence of clinical signs and symptoms of active disease [4]. However, TST and IGRA do not distinguish latent TB from active disease, and neither have high accuracy to predict subsequent active tuberculosis [5]. Better understanding of the biology of Mtb and of LTBI is necessary in order to develop better diagnostic methods and treatment options. However, the interplay between Mtb and the human host is incompletely understood.

Conventionally, LTBI is conceived as Mtb remaining in an inactive, stationary phase in the granuloma as a stable latent population of bacilli capable of surviving under stressful conditions generated by the host [6]. Alternatively, viable non-replicating persistent Mtb reside within alveolar epithelial cells in the lung, with reactivation being associated with the upregulation of resuscitation promoting factors within MTB and the escape of newly dividing microorganisms into alveoli and bronchi [7]. Recent advances in imaging technologies such as computed tomography (CT) combined with positron emission tomography (PET) have aided the evolution of a concept that LTBI encompasses a diverse range of individual states extending from sterilizing immunity in those who have completely cleared the infection via an effective acquired immune response, to subclinical active disease in those who harbor actively replicating bacteria in the absence of clinical symptoms, through to active TB disease with clinical symptoms [8], [9]. Thus, it has been proposed that Mtb infection may be better viewed as a continuous spectrum of immune responses, mycobacterial metabolic activity, and bacillary numbers. In this model the impact of HIV infection can be conceptualized as a shift towards poor immune control, higher mycobacterial metabolic activity, and greater organism load, with subsequently increased risk of progression to active disease [3], [8], [9], [10], [11].

Direct measurement of lesional oxygen tension in rabbits [12], and indirect measurements in non-human primates and humans using hypoxia-sensitive probes demonstrate many TB lesions *in vivo* are hypoxic [13]. Hypoxia is only one of the many different stresses Mtb encounters in the granuloma and *in vitro* and animal models are limited in the extent to which they recapitulate the multifactorial environment created by the host to arrest mycobacterial growth. Nonetheless, many conceptual advances have been achieved in recent years in our understanding of mycobacterial physiology under low oxygen conditions, particularly in the areas of gene regulation, metabolism, and energy homeostasis.

## 2 *M. tuberculosis* and hypoxia: *in vitro* studies of bacterial response and adaptation

The existence of a coordinated and inducible response of Mtb to low oxygen conditions was initially revealed by Wayne and colleagues, culminating in the now widely employed *in vitro* “Wayne” model of hypoxia-induced dormancy [14]. In this system, bacteria grown in liquid medium in sealed tubes with limited head space gradually deplete oxygen supplies, leading to a non-replicating state of persistence (NRP) characterized by reduced metabolism and increased drug tolerance. In this state cellular viability can remain unchanged for weeks to months, with synchronized replication resuming following culture reaeration. The inferred similarities between bacteria grown *in vitro* under hypoxic conditions and clinical cases of latent infection have made the Wayne model a key tool for investigating the molecular basis of mycobacterial dormancy. A key caveat is that many of these studies were performed using laboratory strains of Mtb that have been passaged aerobically over many years, these findings therefore need to be revisited using recent clinical isolates.

### 2.1 Gene regulation, hypoxia sensing

Early work on gene expression analysis of Mtb undergoing hypoxic challenge identified a suite of almost 50 genes that were significantly and consistently upregulated relative to aerobic controls. Further work identified that this regulon was controlled by a transcription factor subsequently named DosR (Dormancy Survival Regulator), the activation of which was mediated through two classic two-component system-type transmembrane sensor histidine kinases, DosS and DosT [15]. Activation of DosS and DosT in turn is still the subject of some debate, however strong evidence suggests they sense cellular redox status and dissolved oxygen concentration, respectively, via their heme prosthetic groups [16]. Genes within the DosR regulon are involved in multiple processes including central metabolism, energy generation and gene regulation; however the majority are of unknown function. Interestingly, despite its dominance of gene expression under hypoxia, multiple studies have demonstrated that genetic inactivation of *dosR* results in a relatively mild loss of viability under hypoxia *in vitro* (2–3 logs decrease in CFUs after 30–50 days incubation) [17], [18], [19] and varying responses *in vivo* in multiple animal models [20]. Further evidence suggests these effects may be dependent on the exact hypoxia model, strain, animal model, and growth media used [17], [18], [20], [21]. Furthermore, upregulation of the DosR regulon is not specific to hypoxic challenge (it is also activated by NO and CO [22], [23]; nor is it uniquely controlled by DosST sensing there is significant cross-talk with other TCS regulons [24]. Nonetheless, DosR and its regulon are modestly upregulated in sputum from active TB cases [25], supporting a role for the dormancy survival response in infection.

Outside of the DosR response, other transcriptional regulators have been identified as playing significant roles under hypoxic conditions, although precise functions have not been determined [25], [26]. Galagan and colleagues used ChIP-Seq data from strains overexpressing various transcription factors to develop a detailed map of regulatory interactions in Mtb under hypoxia [26]. They identified Rv0081, itself part of the DosR regulon, as a major regulatory hub controlling multiple hypoxia-relevant processes. Interestingly, the *Mycobacterium smegmatis* homologue of the Rv0079 - a gene within the same operon as Rv0081 - has also been shown to have functional importance in this bacterium during hypoxia in stabilizing ribosomes in the 70S form, in contrast to the higher order structures seen in many enteric bacteria grown under similar conditions [27]. It is yet to be determined if the same effect is observed in virulent Mycobacteria. Meanwhile, other studies have found that while the DosR regulon is strongly induced at the initiation of anaerobiosis, this level is not maintained throughout longer, sustained periods of hypoxia and upregulation of a separate set of genes, termed the enduring hypoxia response (EHR), appears to dominate at these time points [18], [28]; however both responses appear to interact at the regulatory level [26]. The relevance of the EHR in overall bacterial adaptation to hypoxic conditions has yet to be determined.

The roles of gene regulation at the posttranslational level have also been assessed in hypoxic Mtb, and both proteases and Serine/Threonine Protein Kinases (STPKs) have been found to play functional and essential roles [29], [30]. For example, a regulator of the Mtb Clp protease, Rv2745c, was identified as being required for re-adaptation of hypoxia-challenged Mtb to normoxic conditions: while viability under hypoxia of an Rv2745c null mutant was identical to wild-type, much lower viability of the mutant strain was observed upon reaeration [30]. This data complements other studies showing an enrichment of protease and chaperone related genes during reaeration, relative to those observed under hypoxic conditions [31], [32].

### 2.2 Metabolism

**Fig. 1:**
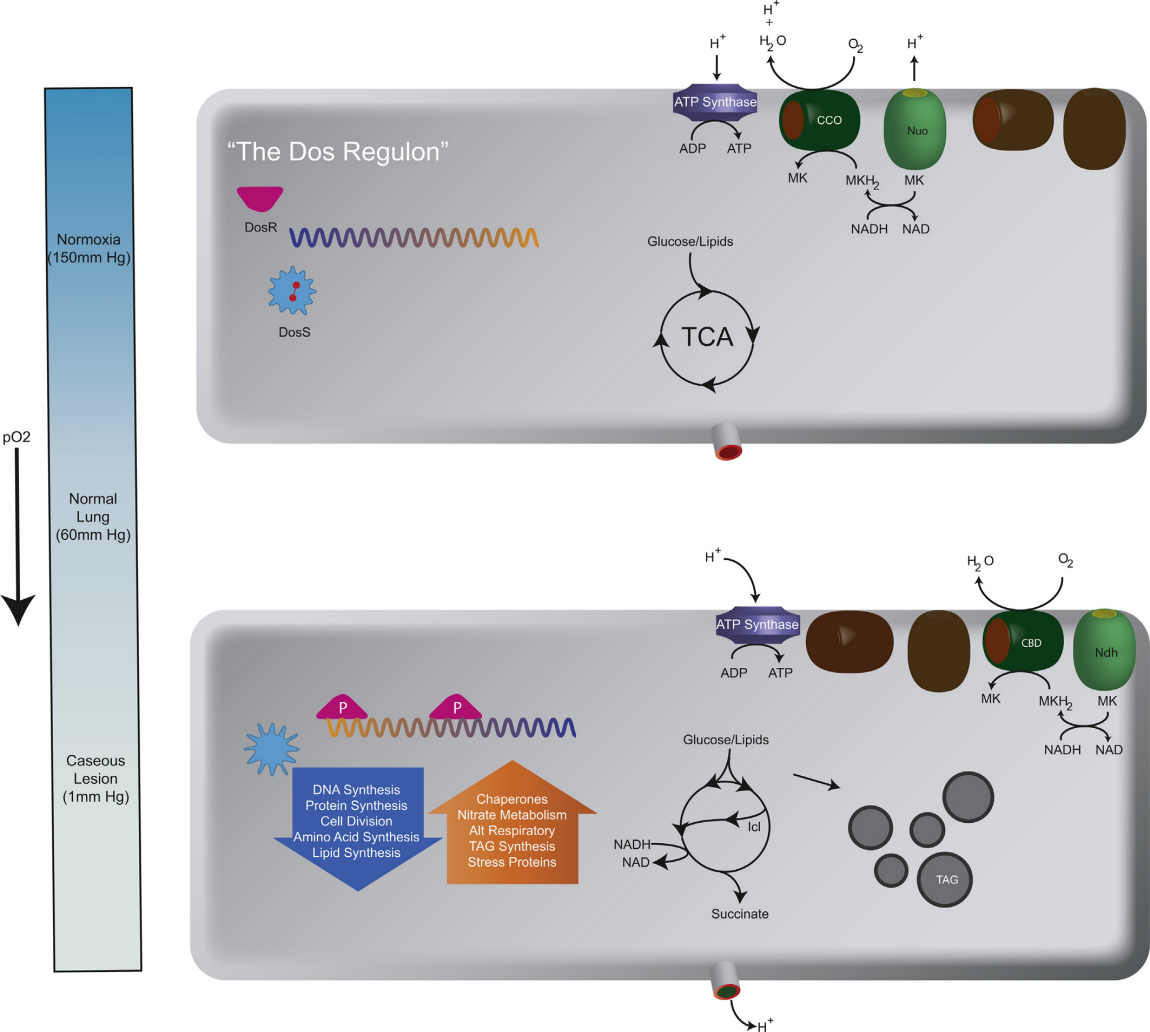
The *M. tuberculosis* response to hypoxia. The top cell depicts the status of several systems involved in key features of the hypoxic response including the status of the DosS/R regulon (off with bound molecular oxygen on DosS in red). The TCA cycle operating normally with glycolytic and lipolytic precursors and components of the terminal electron transport chain operating through the Cytochrome C oxidase (CCO) system and the NADH oxidase Nuo. The bottom cell represents the status of these same systems under hypoxic conditions. DosS has phosphorylated DosR which transcriptionally engages the “DosR” regulon. The TCA cycle operates in a bifurcated cycle through the glyoxylate shunt to reoxidize NADH coupled to the secretion of succinate and intracellular inclusions of triacylglycerols (TAG) accumulate. Reduction of NADH also occurs through the Cytochrome B/D oxidase (CBD) and Ndh which do not pump protons.

A reduction in net carbon flux is a hallmark of hypoxia-induced dormancy in Mtb, suggesting a need to conserve carbon and energy sources for prolonged survival and later resumption of growth when conditions improve. Consistent with the dormancy/hibernation programs of other organisms, Mtb accumulates intracellular triacylglycerides (TAGs) under hypoxia, which correlates with increased expression of the DosR-regulated TAG biosynthetic gene *tgs* [33]. Switching metabolism towards lipid storage may be a major regulator of metabolic slowdown by forcing acetyl-coA flux away from the energy generating catabolic TCA cycle and into anabolic lipid biosynthesis, as evidenced by enhanced metabolic and replication rates of *tgs* mutants in the initial stages of hypoxia relative to wild-type bacteria (Fig. 1) [34]. Also, as previously observed in Mtb grown *in vivo*, upregulation of the isocitrate lyase (*icl*) transcript, protein, and activity levels are also observed under hypoxia [32], [33]. The canonical metabolic role of Icl is to allow growth on fatty acids as the sole carbon source, suggesting a role for Icl in metabolism of stored TAGs as a carbon and energy source under these conditions. This is supported by upregulation of methylcitrate cycle and methylmalonyl CoA pathway genes, enzyme levels, and metabolic intermediates, and the mixed upregulation/essentiality of the gluconeogenic PfkA/B genes during hypoxia and reaeration, consistent with a predominantly fatty acid-based diet [21], [26], [32], [33]. However, under hypoxia Icl appears to have multiple roles, as Icl null mutants grown on glycolytic carbon sources are significantly growth impaired at low oxygen concentrations relative to WT strains [35]. Icl may be involved in conservation of carbon units and/or maintaining optimal NADH/NAD + ratios under the reducing conditions of hypoxia by bypassing the two oxidative and CO_2_ releasing TCA cycle steps, or alternatively in the maintenance of the membrane potential and/or the proton motive force (PMF) via secretion of Icl-produced succinate through a succinate/H^+^ symport system [35], [36]. Indeed, large amounts of succinate are found to accumulate extracellularly in Mtb grown anaerobically and Icl contributes significantly to this effect [35], [36], [37]. Elsewhere, upregulation of other fatty-acid biosynthetic and catabolic genes have been observed in Mtb grown under hypoxia (*fas*, *kasA*, cholesterol catabolism regulation, gluconeogenic *pckA*) [26], [33], [37]. Interestingly, like Icl, many of these genes are also induced upon NO stimulation and *in vivo*, in mouse lung infection, suggesting that a metabolic shift towards lipid metabolism is a general stress response rather than being specific to hypoxia or due to the nature of the provided/available carbon source [33].

Hypoxia-challenged Mtb also substantially down regulate many genes involved in the oxidative direction of the TCA cycle (malate dehydrogenase, citrate synthase, aconitase, putative α-ketoglutarate decarboxylase [33], [37]) and upregulate expression of several members of the reductive direction (fumarate reductase, FR;, PEP carboxykinase and malic enzyme [37]). This suggests a role for the reductive TCA cycle under hypoxia, with fumarate reduction as a fermentative endpoint, and provides an alternative explanation for the observed accumulation of succinate under these conditions [37]. The relative contributions of Icl and fumarate reduction to the production of succinate under hypoxia is debated, but is likely influenced by the available carbon source (i.e. glycolytic vs fatty acid [35], [36], [37]).

Icl catalysis also releases glyoxylate, a metabolite toxic to Mtb cells if left to accumulate. The canonical metabolic fate of glycoxylate is condensation with acetyl-CoA to form malate catalyzed by GlcB, however GlcB activity is down-regulated in hypoxic Mtb [33]. Instead, glycine levels are seen to increase in an Icl-dependent manner [35], [36], inferring subsequent reduction of glyoxylate to glycine as an alternative detoxification step under these conditions. Consistent with this hypothesis, expression and activity of glycine dehydrogenase increases substantially in hypoxic Mtb [14]. Glyoxylate reduction is also a possible fermentative mechanism of regenerating oxidized cofactors during the reductive stress of hypoxia.

Less is known about peripheral metabolic pathways and biosynthesis of other essential compounds and macromolecules under hypoxia. There appears to be growing evidence for shifts in nitrogen metabolism, particularly influenced by the large amount of nitrogen syphoned into the sequestration of glyoxylate (as glycine), changes in glutamine biosynthesis [33], observations of aspartate secretion [36], polyglutamate/glutamine biosynthesis [38], as well as a possible assimilatory role for the DosR-regulated nitrite reductase [39].

### 2.3 Energy generation

Upon entry into hypoxia mycobacteria experience significant decreases in ATP levels and increases in their NADH/NAD + ratio, indicative of a blocked electron transport system (ETS) and consistent with depleted stores of terminal electron acceptors (TEAs) [40]. However, ATP levels remain non-zero throughout hypoxic challenge, and *de novo* ATP synthesis via the ETS (as opposed to via substrate level phosphorylation) is a strict requirement for bacterial survival under these conditions [40]. This suggests that, despite cessation of replication, Mtb maintains both an energized membrane and constitutive ATP production even in the absence of molecular oxygen. Interestingly, transcriptional changes under hypoxia demonstrate a functional switch to the use of less energy efficient respiratory complexes, including upregulation of the non-proton-translocating type II NADH dehydrogenase (*ndh*; essential for survival under hypoxia [40]) and cytochrome bd oxidase (*cydAB*) and down regulation of the proton-pumping type I NADH dehydrogenase (*nuo* [32]). The survival benefit in uncoupling electron transport from generation of the proton motive force (PMF) suggests that cofactor recycling is more important than ATP generation under these conditions, and/or that the PMF is already sufficiently maintained by alternative measures (e.g. succinate, aspartate secretion, nitrate reduction; see previous and later sections).

Succinate dehydrogenase, which physically links the TCA cycle and ETS, has recently been shown to play a key but enigmatic role in mycobacterial adaptation to hypoxic conditions. Genetic deletion of succinate dehydrogenase (SDH) 1 (*sdh-1*; Rv0247c-0249c), the major aerobic SDH, abolishes the ability of bacteria to regulate oxygen consumption (continual high respiratory rates, significantly higher membrane potential relative to wild-type) when approaching hypoxia which subsequently led to increased bacterial death at later stages of anaerobiosis [41]. However, other evidence suggests that Sdh-2 (a homologue of *sdh-1*; Rv3316-3319) may have a key role during hypoxia, either as a canonical succinate dehydrogenase/fumarate reductase [34], [37] and/or in maintenance of the PMF (protonophore treatment of an *sdh-2* null mutant under hypoxia is lethal [41]).

### 2.4 Quinones

While certain proteinaceous modules of the ETS appear to differ between hypo- and normoxia, quinone electron carriers are indispensable across all conditions. Accordingly, inhibition of menaquinone (MQ) biosynthesis is cidal to anaerobic bacteria [16], [42]. Intriguingly, menaquinone:menaquinol (MQ:MQH2) homeostasis under hypoxia may also play a larger regulatory role in addition to electron transport, including in activation of the DosS sensor kinase of the DosR system [16] and regulation of SDH-1 catalytic activity [41]. Also, total MQ pool sizes are reduced under hypoxia, and addition of exogenous MQs lowers cell viability [16], while the degree of saturation of the MQ isoprenyl tail also changes under low oxygen conditions [16], [43]. Deletion of the gene that reduces the MQ isoprenoid side chain results in reduction of efficiency of electron transport and compromised survival in macrophages. The reduced isoprenoid side chain seems highly unlikely to affect the intrinsic redox behavior of this cofactor suggesting that this modification tunes the two forms of MQ to interact with different redox partners and that these therefore have discrete biological functions [44]. Recently, a polyketide synthase (PKS) biosynthetic gene cluster was identified in *M. smegmatis* that was upregulated under hypoxia and coded for the production of novel benzoquinoid compounds. Genetic deletion led to lower viability under hypoxic conditions, which could be rescued upon addition of exogenous synthetic benzoquinones. It is unknown whether Mtb carries the same biosynthetic capabilities. The benefit of such alternative electron carriers under hypoxic conditions is unknown, but may be related to the lower potential difference between oxidized and reduced forms of the benzoquinone moiety relative to the napthoquinone bicyclic ring system of menaquinones [45].

In the absence of molecular oxygen many facultative anaerobes can switch to alternative external TEAs to sustain respiration. Mtb contains all the genetic elements necessary for reduction of nitrate and nitrite, and both of these activities have been detected in growing cells [39], [46]. Nitrite production increases significantly in anaerobically grown Mtb, even though neither expression of the NarGHJI (nitrate reductase) operon nor corresponding catalytic activity in whole cell extracts is significantly different between bacteria grown aerobically or anaerobically. The nitrate import/nitrite export NarK2X operon, however, is part of the DosR regulon and is strongly upregulated under hypoxia [32], [46], suggesting that NarGHJI activity is modified post-translationally following activation of the nitrate import machinery (or directly following oxygen depletion). Interestingly, NarG null mutants display no fitness or viability cost compared to wild-type strains when grown under hypoxic conditions [46], [47], casting doubt on the functional importance of nitrate reduction within the context of the ETS under low oxygen conditions. Similarly, the nitrite reductase NirBD only appears to be expressed and have physiological importance when nitrate or nitrite is supplied as the sole nitrogen source, whether under aerobic or anaerobic conditions [39], [47]. However, adding exogenous nitrate to the growth medium of anaerobic bacteria abolishes the aforementioned succinate secretion, restores ATP levels, lowers the NADH/NAD + ratio, and also buffers against the -cidal effects of mild acid challenge, but only in the presence of an intact NarGHJI operon [35], [47]. Therefore, nitrate reduction may occupy a non-essential but conditionally important role, independent of nitrogen assimilation, in mycobacterial survival of hypoxic challenge by aiding in maintenance of both the PMF (in a similar role to succinate secretion) and ATP levels.

## 3 The host response to hypoxia

### 3.1 Macrophage immune mechanisms during hypoxia

**Fig. 2:**
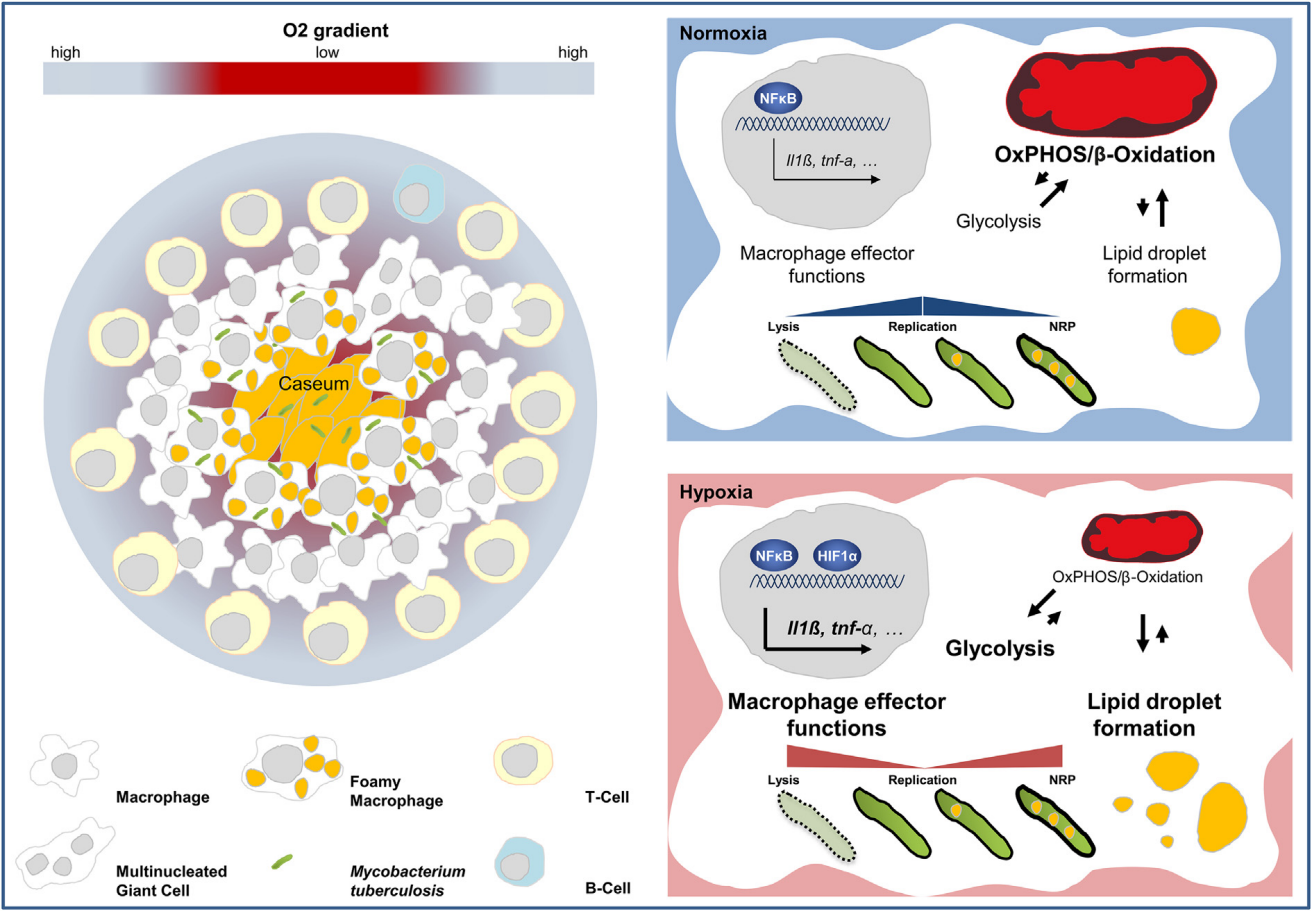
The cellular response to hypoxia. Left panel: Schematic representation of a human granuloma with central necrosis. It is characterized by a decreasing O_2_ tension when getting into the center of a granuloma. Necrotic granulomas are characterized by an outer lymphocyte cuff of T and B cells and a macrophage-rich mid region that surrounds an amorph area of caseum in the center. In these characteristic lesions, mycobacteria often reside within necrotic tissue that has no obvious supply of oxygen. Right panel: Graphic illustration of the *M. tuberculosis* macrophage interaction in normoxia and hypoxia. Infection of macrophages with Mtb leads to a wide array of cellular responses, most of which have been studied under normoxia. Virulent mycobacteria have developed mechanisms operative in infected cells, which allow bacillary replication and persistence by fine-tuning pro- and anti-inflammatory activity. Hypoxic conditions lead to a significnant increase of antimycobacterial effector functions, many of which are significantly enhanced by HIF-1 alpha. This hypoxia-mediated control of Mtb replication is at the same time associated with a significant metabolic reprogramming of its host cell characterized by a shift from oxidative toward glycolytic metabolism. Exposure to hypoxia but also to conserved microbial structures decreased the rate of beta-oxidation, whereas the accumulation of triglycerides increased inside the host cell. This metabolic shift leading to lipid droplet formation is presumably exploited by Mtb. Lipid-laden macrophages are found inside the hypoxic environment of the granuloma and are thought to provide a lipid-rich microenvironment for Mtb, thereby allowing it to adapt to an intracellular lifestyle of non-replicating persistence (NRP) in which it is largely resistant to known bactericidal mechanisms of macrophages and many antimicrobials.

Macrophages undergo substantial phenotypic changes when exposed to reduced oxygen tension and several lines of evidence suggest that hypoxia modulates central effector functions of this key innate immune cell (Fig. 2). The restriction of local oxygen supply was shown to lead to an increased formation of cytokines, chemokines [48], [49] proangiogenic factors [50] but to a reduced eicosanoid synthesis by these cells [51]. Human mononuclear cells and macrophages facing hypoxic conditions secrete significantly enhanced amounts of the major pro-inflammatory cytokines IL-1β and TNF [52], [53]. Various studies have shown that there is a hypoxia-mediated increase in innate immune cell migration into tumor tissue [54] and other hypoxia-related disease settings such as rheumatoid arthritis [55] and atherosclerosis [56]. During migration into inflammatory tissue, monocytes/macrophages encounter a gradual decrease in oxygen availability. The increased migration may be due to a hypoxia-induced chemokine gradient or due to recently observed HIF-1α dependent, chemokine independent accelerated migratory capacity of macrophages, when oxygen tension drops below a certain value [57].

HIF-1α plays a key role for macrophages to adapt to low oxygen tension. Cell-specific deletion of HIF-1α or transient gene silencing in macrophages reduces inflammatory responses with regard to macrophage motility and invasiveness, phagocytic capacity and most importantly bacterial killing [58], [59]. However also under normoxic conditions HIF-1α is induced upon bacterial infection [60]. It plays an important role for the production of key immune effector molecules, including granule proteases, antimicrobial peptides, TNF and nitric oxide (NO). The latter is of major importance since antibacterial immunity critically depends on NO production through Nitric Oxide Synthase-2 (NOS2) in macrophages of infected mice. The importance of HIF-1α for bacteria induced NOS2 expression has been also demonstrated in studies using macrophages stimulated with lipopolysaccharide (LPS) [61], lipoteichoic acid [62] and mycobacteria derived trehalose dimycolate (TDM) [63]. Notably, Mi et al. showed that pattern recognition receptor dependent stimulation of murine macrophages under hypoxia leads to enhanced NOS2 expression when compared to normoxic conditions [64], indicating that cell activation by conserved microbial structures is augmented under hypoxic conditions. Indeed, there is a close relationship between HIF-1α and a central transcriptional regulator for innate immunity and inflammatory processes [65] the transcription factor NF-kappaB (NF-kB) [66], [67], [68]. It was shown that hypoxia itself activates NF-kB through decreased Prolyl hydroxylase-1-dependent hydroxylation of IkappaB kinase-beta [69]. In addition TLR4 activation enhances HIF-1α transcript levels and thus promotes the expression of NF-kB -regulated cytokines in macrophages [70]. The key role of HIF-1α for the production of central immune effector molecules is directly linked to reduction of cellular ATP levels [58]. Under hypoxic conditions HIF-1α promotes the switch to glycolysis so that these cells can continue to produce ATP when oxygen is limited [71]. This change in cellular energy metabolism [72], [73] is also observed in LPS-stimulated macrophages, similar to hypoxic conditions: leading to a metabolic shift towards glycolysis away from oxidative phosphorylation [72], [74].

This phenomenon of aerobic glycolysis in immune cells, resembling the Warburg effect in tumors [75], seems to be necessary for a vigorous and robust response upon classical activation of macrophages (also referred to as M1), though this metabolic transition results in an abating Krebs cycle which is coupled to a less efficient energy production. This reprogramming leads to an increased production of critical metabolites such as succinate, itaconic acid and nitric oxide (NO), all of which have key effector functions during infections [76], [77], [78]. During activation macrophages use other metabolic pathways to satisfy their need for precursor molecules. For example, murine macrophages use an aspartate-arginosuccinate shunt to maintain Interleukin-6 and NO production during M1 activation [79]. Huang et al. showed that cell-intrinsic lysosomal lipolysis is essential for alternative activation (M2) of macrophages [73], further substantiating the link between inflammatory activation and metabolic reprogramming. These studies not only show that inflammatory activation modulates cellular metabolism, but also suggest that the metabolic pathways themselves alter macrophage effector functions dramatically [73], [79]. Intriguingly, the Krebs cycle metabolite succinate serves as an inflammatory signal in macrophages, enhancing IL-1β production by stabilizing HIF-1α [76]. This study, and also the work of Haschemi et al. implicating the carbohydrate kinase-like protein CARKL as an immune modulator in macrophages, shows that metabolic reprogramming is required for full macrophage effector function [80]. However, it also suggests that a manipulation of biosynthetic pathways or changes in metabolite levels may affect immune cell function, as shown for saturated and polyunsaturated fatty acids in dendritic cells [81].

### 3.2 The macrophage/Mtb interaction in hypoxia

Mtb infects macrophages, dendritic cells and neutrophils, with macrophages most extensively studied. Infection with Mtb leads to a wide array of cellular responses, most of which have been studied under normoxia. The evolutionary success of virulent mycobacteria likely depends on cross-species-conserved mechanisms operative in infected cells [82], which allow bacillary replication and persistence by fine-tuning pro- and anti-inflammatory activity [83]. Limited inflammation results in improper activation of macrophages, defective antimicrobial activity, and intracellular survival of the bacilli. Excessive inflammation promotes recruitment of additional Mtb-permissive cells, cell death, and extracellular replication of the bacilli [84]. Most studies indicate that reduced tissue oxygen promotes innate immune cell functions. From a host perspective, by affecting the fine-tuned inflammatory balance within granulomas, hypoxia could do both, either improve the immunity against Mtb, or lead to an impaired growth restriction by causing excessive inflammation and immunopathology.

Human monocyte derived macrophages cultured in 5% oxygen, corresponding to the physiological tissue concentration, permitted significantly less growth than those cultured at the 20% oxygen levels of ambient air [85]. Meylan et al. concluded that macrophages cultured at low oxygen tension may differ from their counterparts cultured at a higher oxygen level in that their intracellular milieu is less supportive of mycobacterial growth. A low pO_2_, which is closer to tissue conditions, did not affect the growth of free-living bacteria but strikingly reduced the growth of intracellular mycobacteria. The growth inhibitory effect was not due to a putative differential response to IFN-γ or TNF-α at low oxygen conditions, but was associated with a shift from oxidative toward glycolytic metabolism, consistent with earlier work in which macrophages cultured at low pO_2_ showed a metabolic shift toward glycolysis [86]. This was an early hint that metabolic changes contribute to Mtb growth control in macrophages. Recent data now show that glycolysis is involved in Mtb growth control in human and murine primary macrophages [87]. A third study also clearly demonstrated significantly decreased growth of Mtb under hypoxia (1% O_2_), when compared to human macrophages kept at 20% [88]. Importantly, macrophage viability, phagocytosis of live Mtb bacteria and Mtb-induced cytokine release were not affected. It has been shown that hypoxia also leads to the induction of autophagy [89], an important mechanism known to limit the growth of intracellular pathogens including Mtb [90]. However there are no data that imply a functional role for this anti mycobacterial effector mechanism under hypoxic conditions. Thus the molecular mechanisms limiting Mtb growth under hypoxic conditions are still incompletely understood. At the same time Mtb is thought to adapt to an intracellular lifestyle of non-replicating persistence (NRP) in which it is largely resistant to known bactericidal mechanisms of macrophages and many antimicrobials [91].

This hypoxia-mediated control of Mtb replication is at the same time associated with a significant metabolic reprogramming of its host cell. Human macrophages cultured for 24 h under hypoxia (1% O_2_) accumulate triacylglycerols (TAG) in lipid droplets [92]. The authors observed increased mRNA and protein levels of adipocyte differentiation-related protein (ADRP) also known adipophilin/perilipin 2, a key factor of lipid droplet formation [92]. Exposure to hypoxia but also to conserved microbial structures decreased the rate of beta-oxidation, whereas the accumulation of triglycerides increased inside the host cell. This phenomenon has recently been attributed to a metabolic switch towards glycolysis [76] by simultaneously decreasing lipolysis and fatty acid oxidation [73]. It appears this metabolic shift leading to lipid droplet formation is exploited by Mtb. Daniel et al. observed that human peripheral blood monocyte-derived macrophages and THP-1 macrophages incubated under hypoxia accumulate Oil Red O-stained lipid droplets containing TAG [93]. The authors were the first to study this effect in the context of Mtb infection. They demonstrated that inside hypoxic, lipid-laden macrophages, nearly half the Mtb population developed phenotypic tolerance to isoniazid, lost acid-fast staining and accumulated intracellular lipid droplets. The fatty acid composition of host and Mtb TAG were nearly identical suggesting that Mtb utilizes host TAG to accumulate intracellular TAG. Other groups suggested that Mtb actively induces this type of lipid-laden phenotype via targeted manipulation of host cellular metabolism resulting in the accumulation of lipid droplets in the macrophage [94]. Mtb oxygenated mycolic acids (MA) trigger the differentiation of human monocyte-derived macrophages into foamy macrophages [95]. Interestingly it has been observed that inhibition of autophagy leads to increased levels of TAG and lipid droplets, and pharmacological induction of autophagy leads to decreased levels of lipid droplets [96]. This may be of functional relevance, since it was shown that Mtb uses a miRNA circuit to inhibit autophagy and promote fatty acid stores in lipid droplets to ensure its own intracellular survival [97]. Lipid-loaded macrophages are found inside the hypoxic environment of the granuloma. They contain abundant stores of TAG and are thought to provide a lipid-rich microenvironment for Mtb [95], [98]. Numerous studies have demonstrated that Mtb relies on fatty acids and also cholesterol as important nutrients during infection, which are used for energy synthesis, virulence factor expression, cell wall and outer membrane construction; and to limit metabolic stress [99], [100], [101], [102], [103]. Moreover, the development of the lipid-rich caseum in the human TB granuloma has been shown to correlate with a realignment in host lipid metabolism within the granuloma, suggesting a pathogen-driven response leading to the pathology necessary for Mtb transmission [104].

### 3.3 The TB granuloma and hypoxia

The formation of granulomas is the hallmark of *Mtb* infection. A granuloma can be defined as an inflammatory mononuclear cell infiltrate that, while capable of limiting growth of Mtb, also provides a survival niche from which the bacteria may disseminate. The tuberculosis lesion is highly dynamic and shaped by both, immune response elements and the pathogen [105]. During disease the formation of necrotic (caseous) granuloma may occur. Necrotic granulomas have an outer lymphocyte cuff dominated by T and B cells and a macrophage-rich mid region that surrounds an amorphous center of caseous necrosis [106]. In these characteristic lesions, mycobacteria often reside within necrotic tissue that has no obvious supply of oxygen [91]. Indirect evidence links changes in oxygen tension with varying TB disease [28]. Intriguingly tuberculosis infections preferentially occur in the most oxygen-rich sites in the human body [107]. In line with these data is the observation that within the lungs of patients failing TB chemotherapy, histological examination of different lung lesions revealed heterogeneous morphology and distribution of acid-fast bacilli [108]. Both studies suggest that reduced levels of O_2_ may limit Mtb growth *in vivo*. It is presumed that Mtb resides in these regions in a slow growing or non-replicating form, due to limited availability and supply of oxygen and nutrients [109].

A number of animal model systems including mice, guinea pigs, rabbits, zebrafish and non-human primates are used to research aspects of granuloma immunopathology in mycobacterial infections. The low dose aerosol model of experimental TB infection in mice has been valuable to define immunological mechanisms of protection against infection, the virulence of mycobacterial strains, or validating novel chemotherapeutic strategies against TB [110], [111]. However mice infected with Mtb fail to produce highly organized caseous or necrotic lesions and do not develop hypoxic regions within their infected lungs [12], [112] suggesting that standard mouse models of persistent tuberculosis may not be suitable for the study of the hypoxic response in Mtb infection. In contrast to mice tuberculous granulomas in guinea pigs, rabbits, nonhuman primates [12], and zebrafish [113] are hypoxic and are appropriate models to study the effect of low oxygen tension in Mtb infection. However three independent, recently developed mouse models may offer new opportunities to study these effects also in TB infected mice. Dermal TB infection of NOS-deficient mice results in development of classic human granuloma pathology when IFN-γ or TNF-α activity is blocked *in vivo* [114]. Unlike BALB/c and C57Bl6 mice, C3HeB/FeJ mice infected with Mtb showed evidence of lesion hypoxia, fibrosis, liquefactive necrosis, and occasional cavity formation [115]. Very recently aerosol Mtb infection of IL-13 overexpressing mice resulted in pulmonary centrally necrotizing granulomas with multinucleated giant cells, a hypoxic rim and a perinecrotic collagen capsule, with an adjacent zone of lipid-rich, acid-fast bacilli-containing foamy macrophages, thus strongly resembling the pathology in human post-primary TB [116]. Thus the use of human tissues or an appropriate animal model to study the host granulomatous response to Mtb is of ultimate importance.

What are the characteristic features of macrophages in hypoxic conditions within the granulomatous lesion? Macrophages in granulomas are both antimycobacterial effector but also the host cell for Mtb. Detailed immunohistochemical analysis of granulomatous lesions from Mtb infected cynomolgus macaques, a non-human primate, using a combination of phenotypic and functional markers suggests that macrophages with anti-inflammatory phenotypes localized to outer regions of granulomas, whereas the inner regions were more likely to contain macrophages with proinflammatory, presumably bactericidal, phenotypes. Active lesions display a gradient of anti- and pro-inflammatory phenotypes, with anti-inflammatory CD163^+^ iNOS^+^ Arg1^high^ macrophages on outer margins and proinflammatory CD11c^+^ CD68^+^ CD163^dim^ iNOS^+^ eNOS^+^ Arg1^low^ macrophages toward the center, thus making it possible to mount antibacterial responses safely away from uninvolved tissue. These data support the concept that granulomas have organized microenvironments that balance antimicrobial and anti-inflammatory responses to limit pathology in the lungs [106]. This is consistent with a recent study demonstrating that inflammatory signaling in human tuberculosis granulomas is spatially organized [117]. The authors applied laser-capture microdissection, mass spectrometry and confocal microscopy, to generate detailed molecular maps of human granulomas. It was observed that the centers of granulomas have a pro-inflammatory environment that is characterized by the presence of antimicrobial peptides, reactive oxygen species and proinflammatory eicosanoids. Conversely, the tissue surrounding the caseum has a comparatively anti-inflammatory signature. If one relates these data to the spatial distribution of local oxygen tension within TB granuloma, there is a nearly perfect concordance between areas of hypoxia, necrosis, and a high degree of proinflammatory activities. In other words the highly hypoxic center is the focus of greatest antimicrobial activity, which is surrounded by an area of reduced proinflammatory activity and gradually increasing oxygen tension. It is of particular interest that foamy macrophages, which are key participants in both sustaining persistent bacteria and contributing to tissue pathology are located mainly in the interface region surrounding central necrosis [95]. As a result of the complex host pathogen interplay foamy macrophages in the interface region may reflect the perfect niche and prime location for Mtb to initiate a new round of infection.

The development of hypoxia is also known to be a stimulus for vascularization [118]. In TB it has been observed that cavitary TB patients presented patterns of low vascularization in the areas of peripheral infiltration, whereas tuberculoma lesions were always surrounded by highly vascularized tissue [119]. This is consistent with the finding that progression to necrosis and caseation is associated with the formation of vascular epithelial growth factor (VEGF) by activated macrophages [120], [121]. Indeed VEGF, a primary mediator of host vascularization, has been found to be induced in human tuberculosis patients [122]. In another smaller study VEGF was postulated as a host marker to differentiate active TB from latent TB infection [123]. A recent study showed that vascularization of zebrafish granulomas was accompanied by macrophage expression of VEGF. Most importantly, treatment of infected animals with a VEGFR antagonist led to dramatic reductions in vascularization and bacterial burdens, demonstrating that a granuloma-induced VEGF-mediated angiogenic program is beneficial to mycobacteria [113]. Taken together, while hypoxia seems host protective at first sight, Mtb may exploit the hypoxia-induced host response to ensure its survival and transmission.

## 4 The acquired immune response to hypoxia-inducible Mtb targets

### 4.1 MTB antigen discovery

Understanding the host immune responses following infection with MTB is essential to help design effective vaccines and identify diagnostic and prognostic immune biomarkers. Antigen discovery efforts have been a core activity in mycobacterial research for several decades, facilitated by the availability of the genome sequence [124]. Antigen discovery approaches include i) the use of algorithms for genome-based prediction of immunodominant epitopes, ii) evaluation of candidate antigens/epitopes for T cell recognition, and iii) understanding the relationship between epitope specificity and the phenotype of the responding T cells. All these approaches rely on the assumption that the antigens of interest are expressed, translated and presented by infected cells, where they are recognized by T cells. While the MTB genome consists of close to 4000 genes, little is known about the MTB antigen repertoire that is actually expressed by the bacilli during infection of human cells. Sequencing the genomes of 21 strains, representative of the global diversity of the MTB complex showed, that the majority of the experimentally confirmed human T cell epitopes had little sequence variation, suggesting they are evolutionarily hyperconserved, implying that MTB might benefit from recognition by human T cells [125].

However, this knowledge is biased by the methods used to experimentally confirm the human T cell epitopes: using IFN-gamma production as a read-out. IFN-gamma is the most established readout of cell mediated immune response assays and a hallmark of the Th1 type cellular immunity [126]. The importance of the Th1 type immunity in controlling MTB infection has been established both in mice and humans [127]. However, it may be an incomplete representation of the cytokine repertoire and functional response of T cells to MTB antigens, and we still do not have a validated immune correlate of protection from TB disease to aid antigen discovery and identification of vaccine candidates. Thus, antigens activating immune cells other than CD4+ and/or CD8+ T cells, producing cytokines other than IFN-gamma are less widely explored [128]. While a number of cytokines and chemokines are being evaluated as alternatives to IFN-gamma, data are still preliminary [129]. Additionally, broadening antigen selection strategies is necessary, such as screening subdominant (cryptic) epitopes, which are not, or only weakly, recognized during natural immunity, but are able to induce immunity and protection against MTB challenge, as demonstrated in mouse models [130].

### 4.2 Mtb biology driven antigen discovery leading to potentially infection stage specific antigens

As indicated above, Mtb can adapt transcriptionally to a wide variety of environmental conditions, such as nutrient depletion, shifts in pH and hypoxia *in vivo*. The hypothesis that genes highly induced under such conditions may also be expressed and available as potential T cell targets has led to the derivation of what are termed infection stage specific MTB genes and thus their cognate antigens.

Amongst the first antigens to be investigated were those of the heat shock response: proteins induced under stress conditions, such as elevations of temperature causing denaturation of proteins during infection [131]. Heat shock proteins assist the survival of MTB but also provide a signal to the immune response. The gene Rv0251c is induced most strongly by heat shock in MTB. It encodes Acr2, a member of the alpha-crystallin family of molecular chaperones. The expression of Acr2 increases within 1 h after infection of monocytes or macrophages, reaching a peak of 18- to 55-fold increase by 24 h of infection *in vitro*. However, a deletion mutant (Δacr2) was unimpaired in log phase growth and persisted in IFN-gamma-activated human macrophages, suggesting that Rv0251c is dispensable. The protein Acr2 is strongly recognized by cattle with early primary *Mycobacterium bovis* infection and also by healthy MTB-sensitized people (LTBI). Interestingly, within the latter group, those with recent exposure to infectious tuberculosis had higher frequencies of Acr2-specific IFN-gamma-secreting T cells than those with more remote exposure, suggesting infection stage-specific immunity to tuberculosis [132].

### 4.3 Infection stage specific T cell responses to TB

Several studies evaluated the above candidate genes, and many were found to encode MTB antigens that induce strong immune responses. One of the most abundant upregulated proteins during hypoxia is the 16 kDa (α-crystallin/Acr, Rv2031c, HspX) protein [133], also a DosR regulated antigen. Attributes of immunodominance, predominant expression during mycobacterial dormancy and species specificity made it a highly attractive candidate for the study of the immune response in humans. Further studies demonstrated it to be immunodominant in both the murine and human systems [134], [135]. The most permissively recognized region was found to be between amino acids 91–110, possibly due to its ability to bind multiple HLA-DR alleles [136].

The finding that the IFN-gamma response to Rv2031c was higher in healthy BCG-vaccinated controls compared to those with extensive untreated tuberculosis led to the speculation that prolonged containment (LTBI) in humans may be contributed to by long-lived Rv2031c-specific cells, able to divide on re-challenge, and thus limit dissemination [137]. This was further investigated by comparing T-cell responses against Rv2031c and the secreted MTB protein Ag85B (Rv1886c) in TB patients and various controls. Gamma interferon responses to Rv2031c were higher in MTB-exposed individuals, with no such differences found against the secreted Ag85B. The term ‘latency antigens’ was coined and suggested that subunit vaccines incorporating latency antigens, as well as recombinant BCG strains expressing latency antigens should be considered as new vaccines against TB [138].

These findings prompted the investigation of the human immune response to other DosR regulon encoded genes, summarized in Ref. [139]. Overall, DosR encoded immunodominant antigens have been termed ‘latency antigens’ due to preferential recognition shown by those with LTBI in terms of a higher IFN-γ response, when compared to those with active tuberculosis [140]. In particular Rv1733c, Rv2029c, Rv2627c and Rv2628c induced strong IFN-gamma responses in skin test positive individuals, suggesting that immune responses against these antigens may contribute to the control of LTBI. The immunogenicity of these (and additional) promising DosR regulon-encoded antigens by plasmid DNA vaccination was also assessed in mice. Strong immune responses could be induced against most, the strongest being Rv2031c and Rv2626c, providing proof-of-concept for studies in mice mimicking LTBI models and their extrapolation to humans for potential new vaccination strategies against TB [141]. A number of comprehensive studies followed, partially summarized in Table 1, which is however by no means exhaustive.

### 4.4 The T cell response to antigens encoded by the genes of the enduring hypoxic response (EHR) of MTB

A detailed analysis of MTB genes that are upregulated during the latent stage of infection was considered a priority to identify new antigenic targets for vaccination strategies [139], [142]. Transcriptional analysis of the hypoxic response at later timepoints led to the identification of 230 genes induced between 4 and 7 days of hypoxia, that were named the enduring hypoxic response (EHR) genes [18]. Analysis of EHR encoded proteins could provide novel T cell targets, with the hypothesis that these genes may be expressed *in vivo* and thereby could be targets of the immune response [28].

In order to relate what is expressed by the bacilli *in vivo* or *in vitro*, to what is recognized by human T cells as antigens, a combined bioinformatic and empirical approach was employed as a novel genome based strategy, to guide the discovery of potential antigens. The fold induction of the top 100 highly induced genes at 7 days of hypoxia, their transcript abundance, population specific MHC class II-peptide binding prediction (ProPred), and a literature search was combined, leading to the selection of 26 candidate genes. Overlapping peptides were used in combination with two readout systems, ELISpot for IFN-γ as well as IL-2. Five novel immunodominant proteins: Rv1957, Rv1954c, Rv1955, Rv2022c and Rv1471, showed responses similar to the immunodominant antigens CFP-10 and ESAT-6 in both magnitude and frequency. These findings revealed that a number of hypoxia-induced genes are potent T-cell targets and therefore offers general support to the important role of hypoxia in the natural course of TB infection. Importantly however, only moderate evidence of infection stage specific recognition of antigens was observed [143].

In light of the above findings, the hypoxia inducible MTB specific proteins absent from the BCG vaccine strains were also evaluated. One region of difference (RD) 2 and two RD11 encoded proteins were identified, that are absent from the commonly used BCG strains (Rv1986) and all *M. bovis* strains including BCG (Rv2658c and Rv2659c), respectively. When compared to the immunodominant molecules ESAT-6 and CFP-10, IFN-gamma responses to the RD11 proteins were inferior in both aTB and LTBI groups. A strong IL-2 recall response to Rv1986 was found in LTBI, targeted at two epitopic regions, containing residues 61–80 and 161–180 [144]. These studies confirmed that genomic knowledge does aide antigen discovery, especially when it is complemented with population specific MHC-class II-peptide prediction analysis, as also shown in a different study later [145]. Additionally, these studies also confirmed that a number of EHR genes are expressed *in vivo* and are potent T-cell targets of the immune response. The results further our understanding of the biology of latent infection and offer general support to the hypoxia hypothesis and its relationship to the natural infection of MTB. While some of these findings did not provide support to the hypothesis of infection stage specific antigen recognition, they support an overlapping immunological spectrum between those with latent and active TB disease as suggested [3], [8]. Whilst hypoxia does characterize granulomas in tuberculosis infection, but it is increasingly appreciated and accepted that even those with active TB disease have a spectrum of lesions, similar to those of the latently infected and it is likely that the hypoxic lesions are present in both clinical states [9], [10], [11]. This has been shown in the cynomolgus macaque model: the fate of individual lesions varies substantially within the same host, suggesting that critical responses occur at the level of each individual lesion, to ultimately determine the clinical outcome of infection in the infected host [146].

## Figures and Tables

**Table 1:** Studies investigating the human immune response to ‘latency antigens’.

First author and year published	Antigens evaluated	Antigen formulations tested	Numbers studied (human/mouse)	Main findings
Leyten et al., 2006 [140]	DosR induced: 25 (selected the most strongly expressed proteins of the DosR regulon; first reference to ‘latency antigens’) Immunodominant: 1 (CFP-10)	Recombinant proteins Peptide pools for CFP-10	TB patients on treatment (n = 11), after treatment (cured TB, n = 9), TST + LTBI n = 23, uninfected healthy controls n = 21, all recruited in The Netherlands.	Latently infected individuals recognized more latency antigens (specifically Rv1733c, Rv2029c, Rv2627c and Rv2628), compared to TB patients, who responded more strongly to CFP-10. These data suggest immune responses against latency antigens may contribute to controlling latent Mtb infection.
Schuck et al., 2009 [147]	Immunodominant: 7 DosR induced: 21 Reactivation-associated: 2 Resuscitation promoting factors (Rpf): 4 Resuscitation-associated: 1 (Rv3407)	Recombinant proteins Overlapping synthetic peptides also for Rv3407	Patients with active TB (aTB, n = 20) and controls with LTBI (n = 22), recruited in Germany	Significantly higher T-cell responses to 7/35 antigens tested in LTBI. T cells specific for Rv3407 were exclusively detected in LTBI. Data support the hypothesis that the latency-associated antigens can be exploited as biomarkers for LTBI.
Black et al., 2009 [148]	Immunodominant: 7 DosR induced: 51	Recombinant proteins	Healthy household contacts (n = 131) recruited from 3 sites (South Africa, Uganda, The Gambia)	Rv1733c was the most commonly recognized DosR regulated antigen.
Gideon et al., 2010 [144]	Immunodominant: 3 EHR induced: 3 species specific (RD11 encoded Rv2568c and Rv2659c; and RD2 encoded Rv1986)	Overlapping synthetic peptides in pools of max 13 peptides per pool. Individual peptides for Rv1986.	Patients with active TB (n = 20), LTBI (n = 29), HIV infected LTBI (n = 19, sampled longitudinally after starting ART), recruited in South Africa.	This study evaluated the antigen specific IL-2 response in parallel with the IFN-gamma response. IFN-gamma responses to the RD11 proteins were inferior compared to the immunodominant molecules, in both aTB and LTBI groups. A strong IL-2 recall response to Rv1986 was found in LTBI.
Reece et al., 2011 [149]	Rv2659c, Rv3407 and Rv1733c, expressed by the recombinant rBCGΔureC::hly vaccine	N/A (Recombinant vaccines were tested)	Mice vaccinated and challenged with MTB Beijing/W isolate	Latency associated antigens expressed in a recombinant vaccine can improve long-term protection against MTB challenge.
Chegou et al., 2012 [150]	118 infection stage specific antigens, including: immunodominant: 8 DosR: 51 Reactivation-associated: 23 Rpf: 5 Starvation-induced: 7 Other stress conditions: 24	Recombinant proteins (n = 112) and Synthetic peptide pools (n = 8, with 6–13 peptides per pool)	TB patients (n = 23) and healthy household controls (HHC, n = 101), recruited in South Africa	The rpfs (Rv0867c, Rv2389c, Rv2450c, Rv1009, Rv1884c) elicited higher IFN-gamma responses in HHCs compared to TB patients, and could differentiate TB from non-TB with area under the curve (AUC) ranging between 0.72 and 0.8.
Gideon et al., 2012 [143]	Immunodominant: 3 EHR induced: 26	Overlapping synthetic peptides in pools of 7–14 peptides per pool.	Patients with active TB (n = 37), LTBI (n = 40), recruited in South Africa.	Only moderate evidence of infection-stage specific antigen recognition was observed using IFN-gamma and IL-2 ELISpot as readout. Data suggest antigens are similarly targets of the immune response in active TB and LTBI, consistent with the view of TB being a spectrum of infection.
Commandeur et al., 2013 [151]	2170 MTB genes investigated in an unbiased Ag discovery approach for *in vivo* expression (IVE) during MTB infection in the lungs of mice. 16 antigens selected, expressed during *in vivo* infection (termed IVE-TB) of all four mouse strains, tested in humans.	Recombinant proteins	4 mouse strains, n = 133 skin test positive control persons and n = 7 TB patients, recruited in The Netherlands and Norway.	The 16 IVE-TB antigens identified were also immunogenic in skin test positive controls, representing TB vaccine candidates and/or TB biomarker antigens.
Sutherland et al., 2013 [152]	21 antigens selected based on the *Black* et al. *2009* and *Chegou* et al. *2012* studies, including immunodominant: 4 reactivation-induced: 6 DosR induced: 9 Starvation-induced: 2	Recombinant proteins for 19 antigens, and synthetic peptide pools for 2 antigens (Rv2659c and Rv2660).	N = 1247 persons, including 262 HIV-TB+, 454 HIV-LTBI+ and 204 HIV-LTBI-, as well as 77 HIV + TB+, 250 HIV + LTBI + recruited from 5 sites (South Africa, Uganda, The Gambia, Ethiopia, Malawi)	Results combined from all sites indicated HIV uninfected TB patients showed lower responses to latency antigens (Rv0569, Rv1733, Rv1735, Rv1737) and the rpf Rv0867, compared to LTBI persons.
Serra-Vidal et al., 2014 [153]	60 recombinant antigens: DosR induced: 6 Reactivation-induced: 12 Rpf: 1 Starvation-induced: 1 Other stress conditions: 6 IVE-TB (based on Commandeur et al.): 34	Recombinant proteins	Patients with TB n = 102, LTBI n = 306, healthy controls n = 97, recruited in Spain.	The DosR induced Rv1733 was the most immunogenic and strongly recognized by LTBI compared to TB patients. The Rpf antigen Rv2389 and Rv2435n from the IVE-TB antigens, were also promising LTBI biomarkers in both short term and long term incubation cultures.
Torres et al., 2015 [154]	12 antigens: immunodominant: 2 EHR induced: 10 DosR: Rv1737	Recombinant protein Rv1737 and synthetic peptides for ESAT-6, CFP-10, Rv0081, Rv0569, Rv2031, Rv0288c, Rv3019c, Rv0826, Rv0849, Rv1986, Rv2659c, Rv2693c, Rv1986.	TST + LTBI with documented TB contact (n = 26) and non-documented TB contact (n = 34), followed up longitudinally on INH treatment, recruited in Mexico.	They show an increase in the proportion of IFN-gamma responders to Rv2031, Rv0849, Rv1986, Rv2659c, Rv2693c and the recombinant Rv1737 protein during IPT, which may represent useful markers to evaluate changes associated with treatment of LTBI.
Coppola et al., 2015 [155]	Evaluation of Rv1733c (as the most promising candidate from the above studies) as a potential vaccine candidate	Recombinant protein Rv1733 and synthetic peptides (p57-84 and HLA-DR3 restricted p63-77)	HLA-DR3 transgenic mice immunized, and infected with H37Rv.	Strong T cell and antibody responses detected, Rv1733 a promising vaccine candidate, even in the form of synthetic peptides.
Arroyo et al., 2016 [156]	6 antigens: immunodominant: fusion protein of ESAT-6 and CFP-10 DosR: 3 (Rv1737, Rv2029, Rv2628) Rpf: 2 (RpfA, RpfD)	Recombinant proteins	Contacts of recently diagnosed TB patients (n = 31), and n = 30 long term LTBI (followed for 5–7 years), recruited in Colombia.	Found significant T cell response to the DosR and Rpf antigens in the long term LTBI, indicating a persistent immune response.

LTBI: latent tuberculosis infection; ART: antiretroviral treatment; TST: tuberculin skin test; RD11: region of deletion 11; IVE: *in vivo* expressed; EHR: Extended hypoxic response; INH: isoniazid.
